# Translation, cultural adaptation and validation of the General Medication Adherence Scale (GMAS) in moroccan patients with type-2 diabetes

**DOI:** 10.1186/s12912-023-01457-9

**Published:** 2023-09-04

**Authors:** Arraji Maryem, Iderdar Younes, Mourajid Yassmine, Guennouni Morad, Boumendil Karima, Korrida Amal, El Khoudri Noureddine, Ifleh Mohamed, Khalis Mohamed, Mohamed Chahboune

**Affiliations:** 1 Higher Institute of Health Sciences, Laboratory of Sciences and Health Technologies, Hassan First University of Settat, Settat, 26000 Morocco; 2 Higher School of Education and Training, Science and Technology Team, Chouaîb Doukkali University of El Jadida, El Jadida, Morocco; 3 High Institute of Nursing Professions and Health Techniques (ISPITS), Agadir, Morocco; 4 Research Laboratory of Innovation in Health Sciences (LARISS), Faculty of Medicine and Pharmacy, Ibn Zohr University, Agadir, Morocco; 5 Faculty of Medicine and Pharmacy, Laboratory of Hematology, Mohammad V University, Rabat, Morocco; 6 Mohammed VI Center of Research and Innovation, Rabat, Morocco; 7 International School of Public Health, Mohammed VI University of Health Sciences, Casablanca, Morocco; 8 Higher Institute of Nursing Professions and Technical Health, Rabat, Morocco; 9 Laboratory of Biostatistics, Clinical, and Epidemiological Research, Faculty of Medicine and Pharmacy, Department of Public Health, Mohamed V University in Rabat, Rabat, Morocco

**Keywords:** GMAS, Adherence, Type-2 diabetes, Validation study, Morocco

## Abstract

**Background:**

The objective of the study was to cross-culturally adapt and validate the General Medication Adherence Scale (GMAS) in patients with type-2 diabetes in Morocco.

**Methods:**

The study was a cross-sectional study conducted between September 12 and October 12, 2022, and included patients with type-2 diabetes from a primary health care network. To measure the different psychometric parameters of the construct, data analysis was performed using SPSS v20. The study was approved by the Moroccan Association for Research and Ethics.

**Results:**

A total of 284 patients were included in the study; the results of the different psychometric parameters were largely acceptable. Indeed, the improvement of the goodness-of-fit of the model in relation to the independence model was evaluated by the comparative fit index (CFI), which was higher than 0.95, as well as the normalized fit index (NFI), which expresses the percentage of the general covariance between the variable demonstrated via the tested model when the null model is taken as reference and was also higher than 0.95 in this study. Additionally, the Tucker Louis Index (TLI) or Unstandardized Fit Index, which measures the increase in goodness of fit when moving from the reference model to the model under study, had a value of > 0.95. The correlations between the items were good; indeed, the Kaiser-Meyer-Olkin (KMO) index was > 0.7. The translated tool presents good internal consistency; thus, Cronbach’s α had a value of approximately 0.804 (> 0.7).

**Conclusions:**

The version of the GMAS tool adapted to the Moroccan context has very acceptable psychometric values. This means that Moroccan researchers and health professionals can use it as an instrument to measure adherence among individuals with type-2 diabetes.

## Introduction

Demographic transitions and lifestyle changes have led to increasing chronic diseases, more so in low- and middle-income countries. These chronic diseases not only have unfavorable outcomes on patients but also increase health care costs [1]. The World Health Organization (WHO) defines chronic diseases as any disease that is characterized by its long-standing and slow evolution, usually resulting in death, apparent disability, or even unhealthy complications [2]. Multimorbidity is especially related to functional incapacity, as well as to apparent frailty, especially in elderly individuals [3]. Some examples of chronic illness that may result in functional decline, debility and/or frailty include cancers, cardiovascular diseases, respiratory disorders, and diabetes [2]. Type-2 diabetes is one of the most complex diseases and is characterized by hyper- or hypoglycemia, accompanied by a significant change in insulin sensitivity or secretion [2]. Although diabetes mellitus is not contagious, it is a public health problem that continues to grow due to its prevalence and related complications [4]. In addition, the health costs attributed to diabetes are constantly rising, reaching 629 million US dollars by 2045 [5], thereby implicating diabetes as a major public health dilemma, which is forecast of being the 7th highest cause of death in the world by 2030 [6]. It is a chronic epidemic condition affecting several countries. It is a major public health problem in China, and its prevalence reached 11.2% among adults in 2017, increasing from 9.7% to 2007 [7]. This is due to a lack of physical exercise, overweight status and even the adoption of an inappropriate diet [8]. A 2016 study from China (N = 15,404) and a 2019 study from Sudan and India (N = 55,639) reported that 80% of patients with diabetes were unable to reach their glycemic goals [9]. In Morocco, the prevalence of diabetes has increased over the years, presenting a major public health concern. According to the Ministry of Health, the national estimated prevalence of diabetes was 10.6% in 2018. In addition, the cost of care with medication nonadherence exceeds $100 billion annually [10]. These widespread chronic diseases have impacted the socioeconomic burden of counties as well as individuals and their families [11]. Type-2 diabetes, which is often diagnosed incidentally during a routine check-up, accounts for 90% of the world’s diabetes population [12]. The condition has serious micro- and macrovascular complications that may develop before the clinical diagnosis of the disease itself. The fundamental goal of self-management of diabetes is controlling blood sugar, maintaining normal or near-normal A1C, lifestyle modifications such as exercise and diet, and adhering to medication regimens to prevent or delay complications. The implementation of these health recommendations is described as therapeutic adherence. [13]. However, nonadherence remains a real problem in patients with chronic conditions, especially those with diabetes mellitus (DM); it deteriorates the patient’s health condition, increases the risk of treatment failure and hospital visits, and finally creates a financial burden to the patient and the health care system. To measure patients’ adherence to their treatments, there are several validated tools to measure medication compliance outside Morocco [14]. Among these tools, we cite The General Medication Adherence Scale (GMAS), which has already been validated in several countries, such as Saudi Arabia [15, 16]. To our knowledge, we do not have such tools validated in our Moroccan context, hence the importance of this work, which comes to fill this gap. Therefore, the objective of our study was to cross-culturally adapt and validate the GMAS for patients with diabetes in Morocco.

## Methods

### Study design

This cross-sectional study was conducted face-to-face from 12 September to 12 October 2022 at primary health care facilities in Morocco where curative and preventive care programs are being provided for individuals with chronic diseases.

The study included adult patients > 18 years of age with prediabetes and/or confirmed diabetes, with or without additional comorbidity, and on diabetic therapy for at least one month prior to study enrollment.

We excluded patients who had planned surgery, those who were pregnant, and those who presented with acute illness(s) requiring urgent medical treatment. All subjects provided informed consent in person, through verbal communication, to avoid problems with reading and comprehension, especially for illiterate patients.

### Patient recruitment

A total of 284 subjects were selected from the primary health care institution registry.

### Presentation of the GMAS tool

The General Medication Adherence Scale (GMAS) is a self-assessment tool consisting of 11 items with a maximum score of 33. A score of ≤ 10 indicates poor adherence, 17–26 indicates partial adherence, 27–29 indicates good adherence, and 30–33 indicates high adherence [16].

Consequently, the cumulative ranking for overall medication adherence is as follows: ⩾ 27 adherent and ≤ 26 nonadherent.

Adherence is further divided into three categories:

(1) nonadherence due to patient behavior (questions 1–5); (2) nonadherence due to comorbidities (questions 6–9); and (3) nonadherence due to cost (questions 10–11).

### GMAS adaptation

The GMAS tool was developed for the first time in 2017 by Naqvi et *al*. for the Urdu language [17]. for our study, we followed the standard operational procedure of the WHO [18] for translation of the instrument from English to Moroccan Arabic.

First, the scale was translated by two translators whose native language is Moroccan Arabic and who are fluent in English. This resulted in two Moroccan Arabic versions of the GMAS at this level. Both versions were presented to the panel and checked for conceptual and cultural equivalence. These two versions were reconciled, and a single Arabic dialect version was formulated and adopted at this level. The research team appointed a reviewer who was efficient in both English and Arabic language to develop the back translation of the instrument. Thus, any disagreements in the translated and back-translated versions were addressed at this stage. The final dialectal version of the tool was tested on 20 subjects. No difficulties were noted, and the Moroccan dialect version of the GMAS was found to be satisfactory.

To adapt the original English version of the GMAS tool to Moroccan culture, a few terminologies were added, replaced, or even moved. This process allowed the development of a version that was easy for patients with type-2 diabetes to understand, thus overcoming all the constraints and ambiguities related to intercultural differences. 2.6. Validity of the GMAS.

To evaluate the reliability of the translated GMAS, it was piloted twice in 20 patients with the questionnaires, which used only 11 items, administered 15 days apart. The delay was sufficient to avoid any influence in relation to the answers to the first questionnaire. Reproducibility is considered “good” if intraclass correlation coefficient (ICC) > 0.4 [19], and the internal consistency between the different items was estimated via Cronbach’s α value, which is considered good for values > 0.7. The response rate to all items was used to measure the acceptability of the instrument.

### Ethical considerations

The study protocol was approved by **RB00012973 Moroccan Association for Research and Ethics IRB #1** [09/REC/22]. Patients were briefed about the study and its objectives; participants provided verbal informed consent.

### Statistical analyses

The values obtained in this study were calculated using SPSS Statistical Package for Social Sciences for Windows (SPSS version 20.0, SPSS Inc., Chicago, IL, USA). Although the homogeneity of variances was performed via Levene’s test, the normality of the data was studied via the Shapiro‒Wilk test. The variables with a normal distribution were averaged (SD). The variables with an abnormal distribution are shown as medians (IQRs). Student’s *t* test was used in the evaluation of independence between qualitative and quantitative variables for two categories, and ANOVA was used in the comparison of variables for three or more categories. Spearman’s coefficients measured the correlation between quantitative variables. For the estimation of the association between categorical variables, the chi-square test was used. A difference was considered significant when the *p* value was less than 0.05. In addition, the floor effects (% of patients who had the lowest score) and ceiling effects (% of subjects who had the highest score) as well as the reliability (reproducibility and internal consistency) were estimated for the psychometric properties evaluated in this Moroccan/Arabic version of the GMAS. To determine the constructs, confirmatory factor analysis (CFA) was performed. To check the possibility of factorizing the data, the Kaiser Meyer Olkin test (KMO > 0.05) with a Bartlett test (p < 0.05) was used [20]. To be included in the CFA, each item must express a communality greater than 0.5. Factors with an Eigen value greater than or equal to 1 were considered. The application of an orthogonal rotation (Varimax) was performed when the correlation between the items was lower than 0.3 [20]. The fit of the model was evaluated via the chi-square test (p < 0.05) and by calculating the root mean square residual (RMSR < 0.08: acceptable fit) [21].

## Results

### Sociodemographic information

Two hundred eighty-four patients with diabetes provided responses, and the average age of the participating patients was 58.24 ± 13.827 years, with extremes of 29 and 91 years. Slightly more than half (53.2%) of the patients were female, 65.5% of the respondents were married, and most respondents (78.5%) were illiterate. Most of the patients were unemployed (91.5%). Almost all patients had medical coverage, provided predominantly by the Medical Assistance Scheme (RAMED), which represented 82.81% of all insured persons (Table 1).

**Table 1:** Sociodemographic information of patients (N = 284)

Sociodemographic characteristics	N	%
**Sex**		
Female	151	53.2
Male	133	46.8
**Place of residence**		
Urban	21	7.4
Rural	263	92.6
**Marital status**		
Single	51	18.0
Married	186	65.5
Widow(er)/Divorced	47	16.5
**Level of education**		
No education	223	78.5
Primary/College/High School	54	19
University	7	2.5
**Employment status**		
Unemployed	260	91.5
Retired	12	4.2
Assets	12	4.2
**Social security coverage**		
Insured	256	90.14
Without insurance	28	9.86
**Type of health insurance**		
CNOPS	30	11.71
CNSS	14	5.46
RAMED	212	82.81

CNOPS: National Fund of Social Welfare Organizations

CNSS: National Social Security Fund

RAMED: Medical Assistance Plan

Approximately 40.5% of the subjects were only taking one antidiabetic medication, with metformin 1000 mg being the most common (37%).

Arterial hypertension was the most common comorbidity (43.7%), followed by dyslipidemia (37.7%). The duration of diabetes diagnosis ranged from 6 to 10 years in 37.7% of the subjects; 58.8% reported self-monitoring blood sugars, and 58.8% stated that they were receiving diabetes education.

### Internal consistency and reproducibility in the pilot study

When calculating the Cronbach’s α values, the results showed a good internal consistency of the tool. Indeed, all Cronbach’s α values ranged from 0.79 to 0.94. The reproducibility measured by intraclass correlation coefficient (ICC) showed values above 0.4 (Table 2).

**Table 2:** Reliability and reproducibility scores of the GMAS Moroccan dialect version (Test-Retest) (N = 20)

The different constructions	Score		Spearman’s Correlation			Interclass Correlation Coefficient		Cronbachα
	Test Mean (SD)*	Retest Mean (SD)*	p	Correlation	P	ICC*	P	
PBNA	19,90(0,44)	19,70(0,73)	0,06	0,54	0,01	0,77	0,00	0,87
ADPB	5,90(0,30)	15,95(0,22)	0,23	0,68	0,00	0,65	0,01	0,79
CRNA	7,65(0,81)	7,75(0,55)	0,30	0,99	0,00	0,90	0,00	0,94
Total	43,45(0,99)	43,40(0,88)	0,77	0,83	0,00	0,70	0,00	0,84

ICC*: Intraclass Correlation Coefficient

SD*: Standard Deviation

PBNA: Patient behavior-related nonadherence

ADPB: Comorbidities and pill burden

CRNA: Cost-related nonadherence

### Factorial validity

The Kaiser‒Meyer‒Olkin (KMO) measure of sampling adequacy had a value of 0.775 (> 0.7), with the addition of a significant Bartlett’s test of sphericity (< 0.01). However, the χ2 value that concerns the null model is equal to 1016.219 with a df value of 55.

Based on the values quoted above, the values of NFI, CFI, TLI, and IFI were all on the order of 1; however, all these values were > 0.95. Taken together, this established the factorial validity of the Moroccan version of the GMAS tool.

### Validity of the known group

A significant association (χ2 = 20.119, p = 0.003) was found between medication adherence status and the presence of comorbidities (i.e., HTN, dyslipidemia and others). The Cramer’s V value of 0.266 ensures a moderate association; likewise, no cell had an expected number < 5, so the results were reliable: this implies that patients without diabetic complications do not tend to adhere to drug treatment. However, there was a significant association (χ2 = 7.503, p = 0.049 < 0.05) between adherence category and prescribed treatment, and Cramer’s V value was 0.163, indicating a moderate association, and no cell had an expected number < 5, so the results were reliable: patients who had a single oral antidiabetic drug (OAD) prescribed tended to be adherent. Thus, our hypotheses were confirmed: monotherapy reinforces adherence, and the presence of comorbidities further reinforces medication adherence by increasing patients’ realization of the consequences. The validity of the known group was thus established (Table 3).

**Table 3:** The impact of sociodemographic factors and diabetic disease-related determinants on adherence to treatment (N = 284)

	≤ 26		≥ 27		P	Cramer’s V
	N	%	N	%		
Place of residence	13		271		0.966	0.002
Rural	12	92.30	251	92.61		
Urban	1	07.70	20	7.39		
Sex	13		271		0.604	0.031
Male	7	53.84	126	46.49		
Female	6	46.16	145	53.51		
Marital status	13		271		0.604	0.031
Single	2	15.38	49	18.08		
Married	8	61.53	178	65.68		
Widow(er)	3	23.07	36	13.28		
Divorced	0	00.00	8	2.95		
Level of education	13		271		0.444	0.115
No education	13	100	210	77.49		
Primary	0	00.00	35	12.91		
College	0	00.00	16	5.90		
High School	0	00.00	3	1.70		
University	0	00.00	7	2.58		
Employment status	13		271		0.533	0.067
Unemployed	13	100	247	91.14		
Retired	0	00.00	12	4.42		
Assets	0	00.00	12	4.42		
Social security coverage	13		271		0.890	0.047
Insured	12	92.30	232	85.60		
Without insurance	1	07.69	39	14.39		
Type of health insurance	13		271		0.433	0.098
CNOPS	0	00.00	30	11.07		
CNSS	0	00.00	14	5.10		
RAMED	13	00.00	227	83.76		
Treatment	13		271		0.049*	0.163
One OAD	5	38.46	110	40.59		
OAD + Insulin	3	23.07	15	5.53		
Two or more OADs	1	07.69	66	24.35		
Insulin alone	4	30.76	80	29.52		
Name of OAD taken	13		271		0.202	0.160
Glimepiride 2 mg	0	00.00	18	6.64		
Gliclazide 60 mg	0	00.00	42	15.49		
Metformin 1000 mg	10	76.92	148	54.61		
Gliclazide + Metformin	0	00.00	33	12.17		
Glimepiride + Metformin	3	23.07	30	11.07		
Associations	13		271		0.090	0.151
Monotherapy	6	46.15	179	66.05		
Dual therapy	2	15.38	83	30.62		
Triple therapy and more	2	15.38	9	3.32		
Associated diseases	13		271		0.003*	0.266
AHT	3	23.07	121	44.64		
Dyslipidemia	4	30.76	113	41.69		
Other pathologies	4	30.76	25	9.22		
AHT + Dyslipidemia	1	07.69	4	1.47		
AHT + Others	1	07.69	1	0.36		
AHT + Dyslipidemia + Others	0	00.00	7	2.58		
Duration of disease	13		271		0.998	0.043
Less than a year	1	07.69	16	5.90		
1 to 5 years	5	38.46	88	32.47		
6 to 10 years	4	30.76	10	38.00		
11 to 15 years	1	07.69	25	9.22		
16 to 20 years	1	07.69	22	8.11		
21 years and more	1	07.69	17	6.27		
Self-monitoring of blood glucose	13		271		0.102	0.127
Yes	4	30.76	167	61.62		
No	9	69.23	117	43.17		
Information about diabetes	13		271		0.127	0.091
Informed patients	5	38.46	162	59.77		
Uninformed patients	8	61.5	109	40.22		

*Significant p value < 0.05

AHT = Hypertension

OAD = oral antidiabetic drug

### The reliability and internal consistency of the GMAS tool (N = 284)

Cronbach’s α = 0.804 (> 0.7) for three constructs (PBNA, ADPB, CRNA), encompassing the 11 items, although the ICC was 0.272 (95% CI: 0.232–0.317); however, the Cronbach’s α values were 0.769, 0.700, and 0.700 for the three constructs, respectively (Table 4).

**Table 4:** Reliability and reproducibility scores of the GMAS Moroccan dialect version (N = 284)

	Interclass Correlation Coefficient		Cronbach α
	ICC*	P	
PBNA	0.39	0.00	0.760
ADPB	0.36	0.00	0.700
CRNA	0.53	0.00	0.700
Total	0.27	0.00	0.804

ICC*: Intraclass Correlation Coefficient

PBNA: Patient behavior-related nonadherence

ADPB: Comorbidities and pill burden

CRNA: Cost-related nonadherence

## Discussion

Diabetes is a chronic condition requiring both routine and complex individual measures [4]. Efficient therapeutic compliance involves developing knowledge and skills through regular monitoring of blood glucose levels to prevent long-term complications, as well as adopting a valuable strategy for improving the health behaviors of these patients with diabetes [4]. Characterization of medication adherence using a self-report tool is the most common, effective and cost-effective way of assessing medication adherence [22]. Various self-assessment tools have been developed, including the Shea Scale [23], the Adherence to Refills and Medications Scale (ARMS) [24], the Morisky Medication Adherence Scale (MMAS) [25] and others. However, many studies have reported that no single tool qualifies as a standard for estimating adherence [22]. GMAS was designed with the shortcomings of the above instruments in mind. The objective of our study was the cross-cultural adaptation and validation of the GMAS tool in the Moroccan context after it was used by Naqvi et al. in Pakistan [17], an English-speaking population (January 2018), Saudi Arabia (April 2018, with the English language) [26] and elsewhere in the Arabic language in December 2019 [16]. The GMAS tool has been translated and cross-culturally validated in the Moroccan context in the present study. Theoretically, the number needed to successfully validate the scale should be in the range of 55–110 patients [27, 28]; however, the present study was able to exceed this figure by collecting data from 284 patients. This sample size was larger than that used in the study conducted in Saudi Arabia [26] and similarly larger than that employed in another study that was able to validate the eight-item Morisky Medication Adherence Scale (MMAS-8) in Saudi Arabia [29]. This aspect could be qualified as a strong point of our study. The factorial validity was estimated using a CFA by calculating suitability indices such as NFI, CFI, TLI, IFI and the KMO measure. These fit indices in our study had a value of 1, and they were in a logical range (> 0.95). The KMO value was 0.775 (> 0.7). The study conducted in Pakistan revealed that these indices, NFI, TLI, and CFI, had the following values: 0.93, 0.93, and 0.97, respectively, with a KMO value of 0.832. For the study conducted in Saudi Arabia in English, the indices had the following values: 0.93, 0.99, and 0.99, respectively, with a KMO value of 0.705. For the assessment of GMAS in Saudi Arabia in Arabic, they had the following values: 0.960, 0.954, and 0.979, respectively, with a KMO value of 0.870. All these above reported values confirmed the goodness of fit of this three-factor model GMAS tool. Convergent validity is established if the value that concerns the average factor loadings is ≥ 0.7 [30]. Our study revealed an average factor of 0.749 for the three constructs, although in the GMAS tool utilized in Pakistan, this loading had values of 0.70, 0.73, and 0.76, respectively, for the three constructs. However, the English GMAS versions used in Saudi Arabia were 0.75, 0.70, and 0.72 for the three constructs, respectively, and the factor loading of the GMAS in Arabic was 0.725 for all constructs in Saudi Arabia. The construct validity of the tool was estimated via the correlation of the adherence score with the pill burden: the number of medications prescribed by the treating physician. To assess the correlation, Spearman’s σ correlation coefficients were calculated, and the validity was established in the case of a correlation coefficient σ ≥ 0.3, with a p value < 0.05 [31–33]. In our study, σ had a value of 0.83, with p < 0.001, while σ for the GMAS in Arabic had a value of 0.388 with p < 0.001 in Saudi Arabia. The reliability of the GMAS tool was assessed through Cronbach’s α. In addition, internal consistency was assessed via the item-total correlation (ITC) and ICC. Our study revealed a Cronbach’s α of 0.804 for the three constructs, which was less than that of the following studies: the Pakistani study, which had a value of 0.819; the Sudanese study [34], which had a value of 0.834; the Saudi study (in Arabic), which had a value of 0.865; and the Vietnam study [9], which had a value of 0.879. However, the Cronbach’s α found in our study is higher than that of the study conducted in Saudi Arabia in English, which had a value of 0.740. The present study encountered a number of limitations that need to be specified: the sensitivity, specificity, and accuracy of the scale were not established, and these three validation modalities would have contributed to the strength of the measurement tool. However, the other validity measures that were developed, combined with the acceptable reliability that was demonstrated, reflect the scale’s suitability for assessing adherence in the target population.

## Conclusion

The Moroccan Arabic-dialect version of the GMAS tool was translated and validated in Moroccan patients with type-2 diabetes and was able to satisfy the validity criteria for the majority of psychometric parameters. As a result, health care professionals caring for patients with diabetes can use this tool to measure adherence. This is important because physicians, nurse practitioners, physician assistants, and even pharmacists manage diabetes patients.

## Data Availability

The datasets used for the present analysis may be made available upon reasonable request by contacting the corresponding author.
